# Intravenous Low-dose Buprenorphine for Acute Pain Management in the Emergency Department: A Case Series

**DOI:** 10.5811/cpcem.43494

**Published:** 2025-07-14

**Authors:** Jonathan Lee, Nicholas Ashenburg, Joshua Park, Terence Ahern

**Affiliations:** * Stanford University School of Medicine, Department of Anesthesiology, Perioperative, and Pain Medicine, Palo Alto, California; † Stanford University School of Medicine, Department of Emergency Medicine, Palo Alto, California; ‡ Stanford Health Care, Department of Pharmacy, Palo Alto, California

**Keywords:** pain, analgesia, buprenorphine, opioids, pain management

## Abstract

**Introduction:**

Buprenorphine is used for treating opioid use disorder, but its role as an analgesic in the emergency department (ED) is frequently overlooked. Emerging evidence indicates that, at low doses, it can be used safely and advantageously as an alternative to full-agonist opioids for treating acute pain.

**Case Series:**

This case series examines the novel use of intravenous (IV) low-dose buprenorphine for acute pain management in the ED for five patients presenting with diverse past medical history and varied painful indications.

**Conclusion:**

Intravenous low-dose buprenorphine may represent an important new tool in our ED armamentarium, and research into its role in emergency pain management is warranted. Further work is needed to determine optimal dosing strategies and identify which patients will be most likely to benefit from IV low-dosebuprenorphine in the ED.

## INTRODUCTION

Buprenorphine is a high-affinity partial μ opioid receptor agonist with unique and complex pharmacology, commonly used for managing pain across various medical specialties.^1^–^3^ When administered at doses lower than those used for opioid use disorder, emerging evidence supports its safety and efficacy as an alternative to full-agonist opioids for treating acute pain.^4^–^5^ Currently, research on low-dose buprenorphine in the emergency department (ED) is limited, and existing studies have primarily focused on sublingual dosing. This case series highlights the novel use of intravenous (IV) low-dose buprenorphine as an alternative to full-agonist opioids for the treatment of acute undifferentiated pain in the ED.

## CASE SERIES

### Patient 1

A 71-year-old male with a history of metastatic pancreatic cancer presented with abdominal pain and elevated liver function tests. On arrival, his vital signs were within normal limits, and he reported severe abdominal pain rated as 10/10 on the numeric rating scale that was intractable to home doses of 5 milligrams (mg) oxycodone. The patient reported using oxycodone intermittently at home for several months but found its pain relief too short-lasting and experienced intolerable gastrointestinal side effects, primarily constipation. He was administered 150 micrograms (μg) of IV low-dose buprenorphine and 10 mg of IV ketorolac while awaiting computed tomography (CT). Labs were notable for leukocytosis of 14.4 x 103 per microliter (K/μL) (reference range: 4.0–11.0 K/μL), hyperbilirubinemia of total bilirubin 2.9 mg/dl (<1.2 mg/dL), and elevated alkaline phosphatase of 693 Units per liter (U/L) (4–30 U/L). The patient experienced significant pain relief, with a reduction of more than five points on the numerical rating scale and no reported side effects on reassessment one hour later. He expressed satisfaction with the treatment. Subsequently, he was found to have pancreatic duct stenosis and was admitted to the oncology service, where IV low-dose buprenorphine doses were continued for pain management.

### Patient 2

A 68-year-old female with a history of multiple surgeries and small bowel obstruction presented with abdominal pain and bloating, rated as 10/10 on the numeric rating scale. Her vital signs, complete blood count, comprehensive metabolic pain, and lactic acid level were within normal limits. Due to her documented intolerance to many full-agonist opioids, she was administered 150 μg IV low-dose buprenorphine and 1000 mg IV acetaminophen while awaiting CT. The patient experienced significant pain relief, reported no side effects, and expressed high satisfaction with the medication on reassessment one hour later. She was diagnosed with a small bowel obstruction and admitted to the surgical service for further management, where her pain regimen was transitioned to full-agonist opioids without issue.

### Patient 3

A 60-year-old female with history of end-stage renal disease and congestive heart failure presented for right hip pain and headache after a ground-level fall. On arrival, her blood pressure was 154/89 millimeters of mercury (mm Hg); otherwise vital signs were within normal limits. Lab testing was unremarkable apart from her baseline chronic kidney disease (creatinine 6.8 mg/dL, blood urea nitrogen 47 mg/dL). She reported severe pain. Given her advanced renal disease, high suspicion for hip fracture, and our preference for a potent, longer acting analgesic, she received 150 μg IV low-dose buprenorphine and 1000 mg IV acetaminophen while awaiting imaging results. The patient experienced significant pain relief, reported no side effects, and expressed high satisfaction with the medication. She was diagnosed with a right femoral neck fracture and admitted to the medicine service for further management, including orthopedic surgery consultation, regional anesthesia consultation, and initiation of an opioid-sparing pain protocol.

### Patient 4

A 44-year-old male with a history of insulin-dependent type 2 diabetes, hypertension, latent syphilis, and prior opioid use disorder presented with multiple abscesses and left facial swelling. He met sepsis criteria, with a temperature of 99.8 ºF, blood pressure of 174/89 mm Hg, respiratory rate of 25 breaths per minute, and heart rate of 99 beats per minute (bpm). He reported facial and neck pain rated as “20 of 10” on the numerical rating scale. Labs were notable for leukocytosis (18.5 K/μL), hyperglycemia (glucose 491 mg/dL), and normal anion gap, renal function, and lactic acid. Due to the patient’s concern regarding his addiction to opioids, he was administered 150 μg IV low-dose buprenorphine and 1000 mg of oral acetaminophen for pain control. The patient experienced significant pain relief with only minor nausea, which was treated with 4 mg of IV ondansetron. He expressed satisfaction with the medication. He was diagnosed with hyperglycemia, sepsis, facial cellulitis, and phlegmon and was admitted to the medical service for management, including broad-spectrum antibiotics and otorhinolaryngology surgery consultation, which resulted in operative management and placement of Penrose drains to treat the infection.

*CPC-EM Capsule*

What do we already know about this clinical entity?

*IV buprenorphine is an μ-opioid partial agonist with potent analgesic effects, FDA-approved for acute pain*.

What makes this presentation of disease reportable?

*IV buprenorphine has been studied extensively perioperatively. This is the first described use in the ED for pain management*.

What is the major learning point?

*(Emergency physicians can use 0.15mg IV buprenorphine to provide safe and efficacious analgesia for acute pain*.

How might this improve emergency medicine practice?

*IV buprenorphine offers a promising alternative to full-agonist opioids, offering another tool for pain management in the ED*.

### Patient 5

A 23-year-old male with a history of chronic back pain following a traumatic injury at age 16 presented with acute worsening of low back pain and bilateral radicular symptoms. On arrival, his vital signs were notable for heart rate of 102 bpm. He reported severe, debilitating pain, 10/10 on the numerical rating scale, and inability to ambulate despite home treatment with nonsteroidal anti-inflammatory medications and tramadol. He was initially treated with 10 mg of IV ketorolac, 1000 mg of IV acetaminophen, 10 mg of oral cyclobenzaprine, and a 5% lidocaine patch. On reassessment 30 minutes later, this regimen had failed to adequately control his pain, and he was subsequently administered 150 μg IV low-dose buprenorphine, which provided excellent relief for the next several hours. The patient reported prior history of opioid use disorder and thus preferred to avoid full-agonist opioid medications, but he was amendable to using low-dose buprenorphine. The patient denied any side effects and expressed high satisfaction with the medication. Labs were unremarkable apart from leukocytosis (white blood cell count 16 K/μL), and magnetic resonance imaging did not show a significant pathologic cause for his pain. He was observed overnight in the clinical decision unit for physical therapy and continued opioid-sparing pain management.

## DISCUSSION

Buprenorphine was approved in the United States in 2002 for the treatment of opioid dependence, significantly improving the safe and effective management of opioid use disorder. However, because of its association with opioid use disorder and classification as a partial μ-opioid receptor agonist, buprenorphine is often misunderstood as a partial analgesic. Its pharmacology is complex and extends beyond its interaction with the μ-opioid receptor; at low-dose ranges, an analgesic ceiling is not known to exist.^9^,^10^ Several studies now suggest that buprenorphine provides analgesia comparable to full-agonist opioids, such as morphine, and offers multiple potential advantages. These include potent analgesia with less euphoria, a ceiling for respiratory depression, lower rates of constipation and pruritus, effective anti-hyperalgesia, reduced immune suppression, and a long duration of action (6–12 hours with a single dose).^8^–^11^ Given these advantages, numerous specialty groups support the use and continued study of buprenorphine for acute pain.^12^–^15^

Low-dose buprenorphine for acute pain involves doses much lower than those used for opioid use disorder. Given the novelty of use in the ED, there is no universally agreed-upon definition for appropriate dose ranges. We recommend IV doses of 150–300 μg. At these doses, low-dose buprenorphine provides potent analgesia with minimal side effects.^5^ To date, very few studies have investigated low-dose buprenorphine in the ED setting. The existing studies assessed sublingual low-dose buprenorphine, comparing it to IV morphine or ketorolac in small groups of patients with specific painful conditions, such as fractures and renal colic.^6^–^8^ These studies found buprenorphine to be an effective analgesic option, although there were slightly higher rates of dizziness and nausea compared to the control treatments when using a 2 mg sublingual dose. This dose equates to approximately 60 morphine milligram equivalents (MME), and we believe it is too high for opioid-naïve or opioid-intolerant patients. Dosing with sublingual formulations comes with challenges, given the lowest dose of generic buprenorphine is 2 mg in the US. Administering this as a half tablet or even quarter tablet is a possibility, but the tablets are small, and because they are not scored for cutting, it could lead to inaccuracies in dosing. Furthermore, this formulation was approved for opioid use disorder, while use for acute pain indication is considered off label.

The IV route of administration for low-dose buprenorphine has not been described in the ED literature, but it may offer an essential new tool in pain management. Intravenous buprenorphine has been approved by the US Food and Drug Administration for use as an analgesic since 1981 and is now available in a generic formulation.^15^ The cost difference of IV buprenorphine compared to IV morphine per MME is marginal. It is available in 150 μg (15 MME) and 300 μg (30 MME) doses, with a rapid onset within minutes and a duration of action up to 6–12 hours. For comparison, a dose of 150 μg is equivalent to 5 mg IV morphine in terms of MME. This makes it titratable and particularly valuable for treating painful conditions expected to last for extended periods.

The patients included in this case series presented with common ED complaints, including abdominal pain, fractures, abscess with cellulitis, and chronic back pain. They may represent ideal candidates for treatment with IV low-dose buprenorphine. Evidence from other specialties suggests that low-dose buprenorphine may be particularly beneficial for acute pain associated with oncologic diseases, renal colic, long bone fractures, neuropathic pain, and in patients with a history of (or active) substance use disorders. However, further research is needed to determine optimal dosing strategies and to identify which patients would benefit from low-dose buprenorphine in the ED.

Our ED has adopted a set of conservative recommendations for the use of low-dose buprenorphine. We recommend against its use in patients on chronic full-agonist opioid therapy. While the risk of precipitated withdrawal is much lower with low-dose buprenorphine, it remains a possibility. Patients who have received a single dose of full agonists (rather than chronic daily use) will not experience precipitated withdrawal with subsequent administration of buprenorphine. However, due to buprenorphine’s high affinity for the μ-opioid receptor, it may displace other full agonists, leading to variable analgesic effects depending on the timing, dose, and potency of the prior opioid. Furthermore, we urge caution in patients with known opioid sensitivity or allergies, as buprenorphine’s potent μ-opioid receptor activity increases the likelihood of cross-reactivity. As research on low-dose buprenorphine evolves, so too will our understanding and its clinical application.

## CONCLUSION

This case series highlights the innovative use of IV low-dose buprenorphine as an alternative to full-agonist opioids for acute pain treatment. Intravenous low-dose buprenorphine may represent an important new tool in our ED armamentarium, and research into its role in emergency pain management is warranted. Further work is needed to determine optimal dosing strategies and identify which patients would be most likely to benefit from IV low-dose buprenorphine in the ED.
